# Associations between serum persistent organic pollutants and polycystic ovary syndrome risk: a case–control study with mediation analysis

**DOI:** 10.3389/fpubh.2026.1807105

**Published:** 2026-06-08

**Authors:** Zekun Zhao

**Affiliations:** Department of Obstetrics and Gynaecology, The University of Hong Kong, Hong Kong, Hong Kong SAR, China

**Keywords:** mediation analysis, mixture analysis, persistent organic pollutants, PFAS, polycystic ovary syndrome, sex hormones

## Abstract

**Background:**

Persistent organic pollutants (POPs), including organochlorine pesticides (OCPs) and per- and polyfluoroalkyl substances (PFAS), are endocrine-disrupting chemicals potentially linked to polycystic ovary syndrome (PCOS). However, evidence on their combined effects and underlying mechanisms remains limited.

**Methods:**

We conducted a case–control study including 146 women with PCOS and 241 controls recruited from the Affiliated Hospital of Hebei University, China (2023–2025). Serum concentrations of 15 POPs (6 OCPs and 9 PFAS) were measured using GC–MS/MS and UPLC-MS/MS. Associations with PCOS were evaluated using multivariable logistic regression, weighted quantile sum (WQS) regression, quantile g-computation (qgcomp), and Bayesian kernel machine regression (BKMR). Mediation analysis examined whether sex hormones mediated these associations.

**Results:**

In single-pollutant analyses, PFOS (OR = 2.63, 95% CI: 1.78–3.88), p,p’-DDE (OR = 2.25, 95% CI: 1.54–3.28), heptachlor (OR = 1.99, 95% CI: 1.26–3.14), and PFHxS (OR = 1.74, 95% CI: 1.15–2.62) were significantly associated with increased PCOS risk. Mixture analyses revealed strong positive associations between the overall POPs mixture and PCOS (WQS OR = 9.29, 95% CI: 3.38–25.50; qgcomp OR = 7.04, 95% CI: 2.79–17.80), with PFOS and p,p’-DDE identified as predominant contributors. BKMR confirmed PFOS (PIP = 1.000) and p,p’-DDE (PIP = 0.998) as the most important contributors. Mediation analysis indicated that testosterone mediated 49.3% of the PFOS–PCOS association, while progesterone mediated 20.9% of the PFOS–PCOS and 24.8% of the p,p’-DDE–PCOS associations.

**Conclusion:**

Exposure to POPs, particularly PFOS and p,p’-DDE, was associated with elevated PCOS risk among Chinese women. Sex hormones, especially testosterone and progesterone, partially mediated these associations, suggesting hormonal disruption as a potential mechanism linking POPs exposure to PCOS.

## 1 Introduction

Polycystic ovary syndrome (PCOS) is the most prevalent endocrine disorder affecting women of reproductive age, with a global prevalence of 10–13% according to the Rotterdam criteria (1, 2). PCOS is characterized by a spectrum of reproductive, metabolic, and psychological complications, including menstrual irregularity, infertility, insulin resistance, and increased risks of type 2 diabetes and cardiovascular disease (3). According to the Global Burden of Disease Study 2021, the worldwide prevalence of PCOS reached 65.77 million cases, representing a substantial public health burden (4). Given its high prevalence and significant health consequences, identifying modifiable risk factors for PCOS is of considerable importance for disease prevention.

The etiology of PCOS is complex and multifactorial, involving both genetic predisposition and environmental factors (5). Although heritability studies suggest a strong genetic component, the rapid increase in PCOS prevalence over recent decades points to significant contributions from environmental exposures (6). Endocrine-disrupting chemicals (EDCs), which interfere with hormonal synthesis, metabolism, and action, have emerged as important environmental risk factors for PCOS (7, 8). Among EDCs, persistent organic pollutants (POPs)—including organochlorine pesticides (OCPs) and per- and polyfluoroalkyl substances (PFAS)—have attracted considerable attention due to their widespread environmental presence, long biological half-lives, and potential to disrupt reproductive endocrine function (9, 10). OCPs such as dichlorodiphenyltrichloroethane (DDT) and its metabolites were extensively used historically and remain detectable in human populations despite regulatory restrictions (11, 12). Similarly, PFAS are ubiquitously detected in human serum worldwide due to their exceptional chemical stability and bioaccumulation properties (13, 14).

Accumulating epidemiological evidence suggests potential associations between POPs exposure and PCOS risk. A large case–control study from China found that plasma concentrations of several PFAS were associated with elevated PCOS risk (15). Recent studies have reported positive associations between PFOS, PFHxS, and increased odds of PCOS among women attending fertility clinics (16, 17). For OCPs, associations between serum concentrations of DDT metabolites and reproductive hormone alterations have been documented, although evidence specifically linking OCPs to PCOS remains limited (18, 19). Sex hormones play a central role in PCOS pathophysiology, with hyperandrogenism and gonadotropin alterations being hallmark features (20, 21). Experimental studies have demonstrated that both OCPs and PFAS can interfere with ovarian steroidogenesis (22, 23), and toxicogenomic analyses have identified steroid hormone biosynthesis as a critical target of PFAS (24). However, whether sex hormones mediate the associations between POPs exposure and PCOS risk in human populations remains largely unexplored.

Despite growing evidence, several important knowledge gaps remain. First, most previous studies focused on either OCPs or PFAS separately, and comprehensive evaluations of combined exposure to multiple POPs classes are lacking. Second, humans are simultaneously exposed to complex chemical mixtures, yet traditional single-pollutant approaches may underestimate cumulative health effects and fail to capture potential interactions among co-occurring pollutants (25, 26). Advanced mixture analysis methods, including Weighted Quantile Sum (WQS) regression, quantile g-computation (qgcomp), and Bayesian Kernel Machine Regression (BKMR), have been developed to address these limitations (27–29). Third, evidence from Chinese populations, where historical OCP contamination and ongoing PFAS exposure remain public health concerns, is particularly limited (30).

To address these gaps, we conducted a case–control study to investigate the associations between serum concentrations of 15 POPs (6 OCPs and 9 PFAS) and PCOS risk among Chinese women. We employed multiple complementary approaches—single-pollutant logistic regression, WQS, qgcomp, and BKMR—to comprehensively evaluate both individual and joint effects of POPs mixtures. Furthermore, we performed mediation analysis to examine whether sex hormones mediate the observed associations between POPs exposure and PCOS. Our findings may provide new insights into the environmental etiology of PCOS and inform public health strategies for preventing this prevalent reproductive disorder.

## 2 Methods

### 2.1 Study design and participants

A hospital-based case–control study was conducted at the gynecology outpatient clinic of the Affiliated Hospital of Hebei University, Baoding, China, from January 2023 to December 2025. Case group: Women aged 18–45 years diagnosed with PCOS according to the Rotterdam criteria (2003) were recruited. PCOS diagnosis required at least two of the following three features: (1) oligo- or anovulation (menstrual cycles >35 days or <8 cycles per year); (2) clinical and/or biochemical hyperandrogenism; (3) polycystic ovarian morphology on ultrasound (≥12 follicles of 2–9 mm per ovary and/or ovarian volume >10 mL). Other etiologies including congenital adrenal hyperplasia, androgen-secreting tumors, Cushing’s syndrome, and thyroid dysfunction were excluded. Control group: Age-matched women with regular menstrual cycles (21–35 days), no signs of hyperandrogenism, and normal ovarian morphology on ultrasound were recruited during the same period. Exclusion criteria for both groups: (1) hormonal medication use within the past 3 months; (2) pregnancy or lactation; (3) history of ovarian surgery; (4) other endocrine disorders; (5) acute or chronic inflammatory diseases; (6) severe hepatic or renal dysfunction. A total of 387 participants (146 PCOS cases and 241 controls) were included in the final analysis. The sample size was determined by the number of eligible participants available during the study period, and all participants who met the inclusion criteria were consecutively recruited.

This study was approved by the Ethics Committee of the Affiliated Hospital of Hebei University (approval no. HDFYLL-KY-2022-107), and written informed consent was obtained from all participants.

### 2.2 Data collection

Demographic characteristics, lifestyle factors, and reproductive history were collected through face-to-face interviews using a standardized questionnaire. Information included age, height, weight, education level, marital status, occupation, monthly income, smoking status, alcohol consumption, age at menarche, menstrual cycle length, gravidity, and parity. Fasting venous blood samples (10 mL) were collected between 8:00–10:00 a.m. during the early follicular phase (days 2–5) for women with regular cycles, or at any time for amenorrheic women after excluding pregnancy. Blood samples were collected into polypropylene tubes to minimize PFAS contamination, centrifuged at 3,000 rpm for 10 min at 4 °C within 2 h of collection, and serum was stored at −80 °C until analysis.

### 2.3 Laboratory analysis

#### 2.3.1 Measurement of persistent organic pollutants

Serum concentrations of 15 POPs were measured, including 6 organochlorine pesticides [OCPs: p,p’-dichlorodiphenyldichloroethylene (p,p’-DDE), p,p’-dichlorodiphenyltrichloroethane (p,p’-DDT), o,p’-dichlorodiphenyltrichloroethane (o,p’-DDT), hexachlorobenzene (HCB), *β*-hexachlorocyclohexane (β-HCH), and heptachlor) and 9 per- and polyfluoroalkyl substances (PFAS: perfluorooctanoic acid (PFOA), perfluorooctane sulfonate (PFOS), perfluorohexane sulfonate (PFHxS), perfluorononanoic acid (PFNA), perfluorodecanoic acid (PFDA), perfluoroundecanoic acid (PFUnDA), perfluorododecanoic acid (PFDoDA), perfluorobutane sulfonate (PFBS), and perfluoroheptanoic acid (PFHpA)].

OCPs and PFAS were analyzed using two separate analytical platforms owing to their distinct physicochemical properties. Isotope-labeled internal standards were used for quantification of each analyte.

Determination of OCPs. Serum OCPs were measured by gas chromatography coupled with triple quadrupole mass spectrometry (GC–MS/MS; Agilent 7890B GC system equipped with an Agilent 7000D triple quadrupole mass spectrometer, Agilent Technologies, Santa Clara, CA, United States). For sample preparation, a 500 μL aliquot of serum was spiked with 20 μL of isotope-labeled internal standard mixture (^13^C₁₂-p,p’-DDE, ^13^C₁₂-p,p’-DDT, ^13^C₆-HCB, and ^13^C₆-*β*-HCH; Cambridge Isotope Laboratories, Andover, MA, United States) and subjected to liquid–liquid extraction with 3 mL of n-hexane/dichloromethane (4:1, v/v). The mixture was vortexed for 2 min and centrifuged at 3,000 rpm for 5 min. The upper organic layer was collected, and the extraction was repeated twice. The combined extracts were passed through an anhydrous sodium sulfate column for dehydration, concentrated to near dryness under a gentle stream of nitrogen, and reconstituted in 100 μL of n-hexane for injection.

Chromatographic separation was performed on an HP-5 ms Ultra Inert capillary column (30 m × 0.25 mm i.d., 0.25 μm film thickness; Agilent Technologies). Helium (purity ≥ 99.999%) was used as the carrier gas at a constant flow rate of 1.0 mL/min. The oven temperature program was as follows: initial temperature 80 °C (held for 1 min), ramped at 20 °C/min to 180 °C (held for 1 min), then at 5 °C/min to 280 °C (held for 5 min), and finally at 10 °C/min to 300 °C (held for 3 min). The injection volume was 1 μL in splitless mode with an inlet temperature of 280 °C. The mass spectrometer was operated in electron ionization (EI) mode at 70 eV with multiple reaction monitoring (MRM). The transfer line temperature was set at 280 °C, and the ion source temperature was maintained at 230 °C. Two MRM transitions were monitored for each analyte: the quantifier transition for quantification and the qualifier transition for confirmation.

Determination of PFAS. Serum PFAS were measured by ultra-performance liquid chromatography coupled with tandem mass spectrometry (UPLC-MS/MS; Waters ACQUITY UPLC system equipped with a Xevo TQ-S triple quadrupole mass spectrometer, Waters Corporation, Milford, MA, United States). A 200 μL aliquot of serum was spiked with 20 μL of isotope-labeled internal standard mixture (^13^C₄-PFOS, ^13^C₄-PFOA, ^13^C₂-PFHxS, and ^13^C₅-PFNA; Wellington Laboratories, Guelph, ON, Canada) and mixed with 400 μL of acetonitrile for protein precipitation. The mixture was vortexed vigorously for 2 min, followed by ultrasonication for 10 min, and then centrifuged at 12,000 rpm for 10 min at 4 °C. The supernatant (400 μL) was transferred to a new tube, diluted with 400 μL of ultrapure water, vortexed briefly, and filtered through a 0.22 μm PVDF syringe filter into a polypropylene autosampler vial for injection.

Chromatographic separation was achieved on a Waters ACQUITY UPLC BEH C18 column (2.1 mm × 100 mm, 1.7 μm) maintained at 40 °C. The mobile phase consisted of (A) 10 mM ammonium acetate in water and (B) methanol. A gradient elution program was used: 0–0.5 min, 30% B; 0.5–3.0 min, 30–90% B; 3.0–5.0 min, 90% B; 5.0–5.1 min, 90–30% B; 5.1–7.0 min, 30% B. The flow rate was 0.4 mL/min and the injection volume was 5 μL. The mass spectrometer was operated in electrospray ionization (ESI) negative mode with multiple reaction monitoring (MRM). Source parameters were optimized as follows: capillary voltage, 2.5 kV; source temperature, 150 °C; desolvation temperature, 500 °C; desolvation gas flow, 1,000 L/h; cone gas flow, 150 L/h.

Quality assurance and quality control. All analyses were performed in a dedicated clean laboratory with HEPA-filtered air to minimize contamination. Procedural blanks (ultrapure water processed identically to samples) were analyzed with each batch of 20 samples to monitor background contamination. Calibration curves were constructed using matrix-matched standards at 8 concentration levels, with correlation coefficients (r^2^) > 0.99 for all analytes. Quality control (QC) samples, prepared by spiking pooled serum with known concentrations of analytes at low, medium, and high levels, were analyzed in duplicate with each batch. The recoveries ranged from 80 to 120% for OCPs and 85 to 115% for PFAS, and the intra- and inter-batch coefficients of variation (CVs) were < 15% and < 20% for OCPs, and < 10% and < 15% for PFAS, respectively.

The limits of detection (LOD) were calculated as three times the standard deviation of procedural blanks or the lowest calibration standard with a signal-to-noise ratio ≥ 3, whichever was higher. The LODs for OCPs ranged from 0.5 to 15.0 ng/g lipid, while those for PFAS ranged from 0.01 to 0.10 ng/mL (Table 1). The detection rates for all analytes ranged from 87.3 to 100%. Concentrations below the LOD were imputed with LOD/√2 for statistical analysis.

**Table 1:** Distribution of persistent organic pollutants concentrations among study participants.

Chemical	LOD	Detection rate (%)	Total median (P25-P75)	Control median (P25-P75)	PCOS median (P25-P75)	*p* value
OCPs (ng/g lipid)						
p,p’-DDE	15	100	183.14 (118.22–307.00)	158.42 (110.32–251.48)	246.31 (138.99–398.77)	<0.001
p,p’-DDT	3	94.1	7.94 (4.90–12.19)	8.52 (5.14–12.75)	7.32 (4.51–11.49)	0.029
o,p’-DDT	2.5	89.9	4.87 (3.35–7.27)	4.75 (3.47–6.82)	5.21 (3.17–7.90)	0.479
HCB	8	98.4	25.97 (18.26–36.67)	26.52 (18.99–36.46)	24.35 (17.37–37.30)	0.307
β-HCH	4	96.6	11.89 (8.37–17.88)	11.67 (8.41–17.64)	12.25 (8.22–17.97)	0.662
Heptachlor	3.5	98.4	10.50 (7.00–14.95)	10.03 (6.76–13.97)	11.78 (8.54–16.17)	0.001
PFAS (ng/mL)						
PFOA	0.5	100	3.06 (2.04–4.35)	2.81 (2.03–4.16)	3.24 (2.20–4.85)	0.085
PFOS	1	100	10.24 (6.75–16.22)	8.89 (6.13–14.42)	12.48 (8.11–19.19)	<0.001
PFHxS	0.4	99.7	1.89 (1.31–2.84)	1.76 (1.24–2.55)	2.33 (1.55–3.35)	<0.001
PFNA	0.3	99.7	1.28 (0.90–1.85)	1.24 (0.86–1.85)	1.34 (0.95–1.85)	0.246
PFDA	0.15	96.6	0.39 (0.26–0.57)	0.38 (0.26–0.55)	0.41 (0.28–0.59)	0.101
PFUnDA	0.12	97.7	0.31 (0.22–0.43)	0.30 (0.22–0.41)	0.32 (0.23–0.45)	0.192
PFDoDA	0.05	92.2	0.10 (0.07–0.13)	0.09 (0.07–0.13)	0.10 (0.07–0.13)	0.464
PFBS	0.08	87.3	0.13 (0.10–0.17)	0.12 (0.09–0.17)	0.13 (0.10–0.18)	0.371
PFHpA	0.12	87.9	0.22 (0.16–0.31)	0.22 (0.16–0.31)	0.22 (0.15–0.32)	0.807

Concentrations are presented as median (P25-P75). OCPs and PFAS concentrations are expressed as ng/g (lipid) and ng/mL. Values below the LOD were imputed with LOD/√2. *p* values were calculated using Mann–Whitney U test. LOD, limit of detection; OCPs, organochlorine pesticides; PFAS, per- and polyfluoroalkyl substances; DDE, dichlorodiphenyldichloroethylene; DDT, dichlorodiphenyltrichloroethane; HCB, hexachlorobenzene; HCH, hexachlorocyclohexane; PFOA, perfluorooctanoic acid; PFOS, perfluorooctane sulfonate; PFHxS, perfluorohexane sulfonate; PFNA, perfluorononanoic acid; PFDA, perfluorodecanoic acid; PFUnDA, perfluoroundecanoic acid; PFDoDA, perfluorododecanoic acid; PFBS, perfluorobutane sulfonate; PFHpA, perfluoroheptanoic acid.

Lipid determination and unit expression. Serum total cholesterol and triglycerides were measured enzymatically on a Beckman Coulter AU5800 clinical chemistry analyzer. Total lipid concentrations were calculated using the Phillips formula: Total lipid (g/L) = 2.27 × total cholesterol (g/L) + triglycerides (g/L) + 0.623. OCP concentrations were normalized to serum lipid content and expressed as ng/g lipid. PFAS concentrations were expressed as ng/mL, as PFAS bind primarily to serum proteins rather than lipids. Detailed MRM parameters for OCPs and PFAS, internal standard information, and detection limits are provided in Supplementary Tables S1–S3.

#### 2.3.2 Measurement of sex hormones

Serum levels of follicle-stimulating hormone (FSH), luteinizing hormone (LH), estradiol (E2), total testosterone (T), prolactin (PRL), and progesterone (P) were measured using electrochemiluminescence immunoassay (ECLIA) on a Roche Cobas e601 analyzer. The intra- and inter-assay CVs were < 5% and < 8%, respectively.

### 2.4 Covariates

Potential confounders were selected based on prior knowledge of PCOS risk factors and determinants of POPs exposure. The following covariates were included in multivariable models: age (continuous, years), body mass index (BMI, continuous, kg/m^2^, calculated as weight/height^2^), education level (categorical: high school or below, college/university, graduate or above), marital status (unmarried/married), occupation (student, unemployed, employed, other), smoking status (yes/no, defined as currently smoking ≥1 cigarette/day), alcohol consumption (yes/no, defined as drinking ≥1 time/week in the past year), monthly income (categorical), age at menarche (continuous, years), gravidity (number of pregnancies), and parity (number of deliveries). Age and BMI were included as they are associated with both PCOS risk and POPs body burden. Socioeconomic factors (education, occupation, income) were included as they may influence dietary habits and environmental exposures. Lifestyle factors (smoking, alcohol consumption) were included as they may affect POPs metabolism and hormonal status. Reproductive history variables (menarche age, gravidity, parity) were included as potential confounders of both POPs accumulation and PCOS risk.

### 2.5 Statistical analysis

Continuous variables were presented as mean ± SD for normally distributed data or median (25th-75th percentile) for skewed data, and compared between groups using independent t-test or Mann–Whitney U test, respectively. Categorical variables were presented as frequencies (percentages) and compared using chi-square test. POPs concentrations were natural log-transformed to approximate normal distributions before regression analyses.

Multivariable logistic regression models were used to estimate odds ratios (ORs) and 95% confidence intervals (CIs) for the associations between individual POPs and PCOS risk. ORs were calculated per unit increase in ln-transformed POPs concentration, adjusting for age, BMI, education, marital status, occupation, smoking, alcohol consumption, income, menarche age, gravidity, and parity. Results were visualized using forest plots.

Given that humans are exposed to multiple POPs simultaneously, three complementary approaches were employed to evaluate the joint effects of POPs mixtures on PCOS risk. Weighted Quantile Sum (WQS) regression was used to estimate the overall mixture effect and identify the relative contribution of each chemical. POPs concentrations were divided into quartiles, and a weighted index was calculated with weights constrained to be non-negative and sum to one. The model was fitted with 500 bootstrap samples, and the direction was constrained to positive based on the hypothesis that POPs exposure increases PCOS risk. Chemicals with weights exceeding 1/15 (0.067) were considered important contributors. Quantile g-computation (qgcomp) was used as an alternative approach that estimates the effect of simultaneously increasing all exposures by one quantile, allowing both positive and negative weights without directional constraint. Bayesian Kernel Machine Regression (BKMR) was used to flexibly model potentially non-linear exposure-response relationships. Posterior inclusion probabilities (PIPs) were calculated to evaluate the relative importance of each chemical, with PIP > 0.5 considered indicative of an important contributor. Univariate exposure-response functions were estimated while holding other chemicals at their median values, and the overall mixture effect was assessed by comparing PCOS risk when all chemicals increased from the 25th to 75th percentile. The model was fitted using 10,000 Markov Chain Monte Carlo iterations with a Gaussian kernel. All mixture models were adjusted for the same covariates. Analyses were performed using the “gWQS”, “qgcomp”, and “bkmr” R packages.

Mediation analysis was conducted to examine whether sex hormones mediate the associations between POPs and PCOS. POPs that were significantly associated with PCOS in single-pollutant analysis (p,p’-DDE, PFOS, PFHxS, and Heptachlor) were selected as exposures, and six sex hormones (FSH, LH, E2, T, PRL, and P) were examined as potential mediators. For each exposure-mediator-outcome combination, the average causal mediation effect (ACME, indirect effect), average direct effect (ADE), total effect, and proportion mediated were estimated using the R package “mediation” with the quasi-Bayesian Monte Carlo method (500 simulations). A mediation effect was considered significant only when all three components (ACME, ADE, and total effect) reached statistical significance (*p* < 0.05), ensuring robust conclusions. All statistical analyses were performed using R software (version 4.2.0). Two-sided *p* < 0.05 was considered statistically significant.

## 3 Results

### 3.1 Characteristics of study participants

A total of 387 women were enrolled in this study, including 146 women diagnosed with PCOS and 241 healthy controls. The demographic and clinical characteristics of study participants are summarized in Table 2. Women with PCOS were significantly younger than controls (25.9 ± 3.6 vs. 27.8 ± 4.1 years, *p* < 0.001) and had higher body mass index (BMI) (25.3 ± 3.9 vs. 22.2 ± 3.0 kg/m^2^, *p* < 0.001). No significant differences were observed between the two groups regarding education level, marital status, occupation, monthly income, smoking status, or alcohol consumption (all *p* > 0.05).

**Table 2:** Demographic and clinical characteristics of study participants.

Variable	Total	Control (*n* = 241)	PCOS (*n* = 146)	*p* value
Age (years)	27.1 ± 4.0	27.8 ± 4.1	25.9 ± 3.6	<0.001
BMI (kg/m^2^)	23.4 ± 3.7	22.2 ± 3.0	25.3 ± 3.9	<0.001
Education				0.078
High school or below	81 (20.9%)	49 (20.3%)	32 (21.9%)	
College/University	210 (54.3%)	123 (51.0%)	87 (59.6%)	
Graduate or above	96 (24.8%)	69 (28.6%)	27 (18.5%)	
Marital status				0.095
Married	234 (60.5%)	154 (63.9%)	80 (54.8%)	
Unmarried	153 (39.5%)	87 (36.1%)	66 (45.2%)	
Occupation				0.47
Professional/Technical	111 (28.7%)	73 (30.3%)	38 (26.0%)	
Service/Sales	155 (40.1%)	91 (37.8%)	64 (43.8%)	
Other	121 (31.3%)	77 (32.0%)	44 (30.1%)	
Monthly income (CNY)				0.827
<5,000	141 (36.4%)	86 (35.7%)	55 (37.7%)	
5,000–10,000	140 (36.2%)	90 (37.3%)	50 (34.2%)	
>10,000	106 (27.4%)	65 (27.0%)	41 (28.1%)	
Smoking status				1
No	331 (85.5%)	206 (85.5%)	125 (85.6%)	
Yes	56 (14.5%)	35 (14.5%)	21 (14.4%)	
Alcohol consumption				0.548
No	307 (79.3%)	194 (80.5%)	113 (77.4%)	
Yes	80 (20.7%)	47 (19.5%)	33 (22.6%)	
Age at menarche (years)	12.9 ± 1.5	12.9 ± 1.4	12.9 ± 1.5	0.869
Menstrual cycle length (days)	29.0 (26.0–34.5)	28.0 (26.0–29.0)	38.0 (31.2–46.0)	<0.001
Gravidity	1.0 (0.0–2.0)	1.0 (0.0–2.0)	1.0 (0.0–1.0)	0.043
Parity	0.0 (0.0–1.0)	0.0 (0.0–1.0)	0.0 (0.0–1.0)	0.228
FSH (mIU/mL)	6.8 (5.6–8.4)	6.9 (5.7–8.5)	6.5 (5.4–8.3)	0.121
LH (mIU/mL)	7.2 (5.4–11.1)	6.1 (4.9–7.5)	11.8 (9.0–16.3)	<0.001
E2 (pg/mL)	49.0 (36.9–60.7)	50.1 (40.6–61.1)	45.3 (34.2–56.7)	0.005
Testosterone (ng/mL)	0.5 (0.3–0.8)	0.4 (0.3–0.5)	0.9 (0.6–1.3)	<0.001
Prolactin (ng/mL)	16.5 (12.5–21.7)	15.1 (11.9–18.8)	19.8 (14.6–25.7)	<0.001
Progesterone (ng/mL)	0.8 (0.6–1.1)	1.0 (0.7–1.2)	0.6 (0.4–0.8)	<0.001

Data are presented as mean ± SD for normally distributed continuous variables, median (P25-P75) for skewed continuous variables, and *n* (%) for categorical variables. *p* values were calculated using independent t-test for normally distributed variables, Mann–Whitney U test for skewed variables, and chi-square test for categorical variables. BMI, body mass index; FSH, follicle-stimulating hormone; LH, luteinizing hormone; E2, estradiol; PRL, prolactin; PCOS, polycystic ovary syndrome.

With respect to reproductive characteristics, women with PCOS exhibited significantly longer menstrual cycle length compared with controls [38.0 (31.2–46.0) vs. 28.0 (26.0–29.0) days, *p* < 0.001]. Age at menarche and parity were comparable between the two groups (*p* > 0.05). As expected, women with PCOS displayed characteristic hormonal profiles, with significantly elevated serum levels of luteinizing hormone (LH) [11.8 (9.0–16.3) vs. 6.1 (4.9–7.5) mIU/mL], testosterone [0.9 (0.6–1.3) vs. 0.4 (0.3–0.5) ng/mL], and prolactin [19.8 (14.6–25.7) vs. 15.1 (11.9–18.8) ng/mL], along with decreased levels of estradiol [45.3 (34.2–56.7) vs. 50.1 (40.6–61.1) pg./mL] and progesterone [0.6 (0.4–0.8) vs. 1.0 (0.7–1.2) ng/mL] (all *p* < 0.01, Table 2).

### 3.2 Distribution of POPs concentrations

The detection rates and distributions of 15 POPs (6 OCPs and 9 PFAS) in serum samples are presented in Table 1. Detection rates ranged from 87.3% (PFBS) to 100% (p,p’-DDE, PFOA, and PFOS), indicating widespread exposure to these compounds in our study population.

Among the OCPs, p,p’-DDE exhibited the highest median concentration [183.14 (118.22–307.00) ng/g], followed by HCB [25.97 (18.26–36.67) ng/g] and *β*-HCH [11.89 (8.37–17.88) ng/g]. For PFAS, PFOS showed the highest median concentration [10.24 (6.75–16.22) ng/mL], followed by PFOA [3.06 (2.04–4.35) ng/mL] and PFHxS [1.89 (1.31–2.84) ng/mL].

Comparing concentrations between groups, women with PCOS had significantly higher serum levels of p,p’-DDE [246.31 (138.99–398.77) vs. 158.42 (110.32–251.48) ng/g, *p* < 0.001], PFOS [12.48 (8.11–19.19) vs. 8.89 (6.13–14.42) ng/mL, *p* < 0.001], PFHxS [2.33 (1.55–3.35) vs. 1.76 (1.24–2.55) ng/mL, *p* < 0.001], and heptachlor [11.78 (8.54–16.17) vs. 10.03 (6.76–13.97) ng/g, *p* = 0.001] compared with controls. Interestingly, p,p’-DDT concentrations were slightly lower in the PCOS group than in controls [7.32 (4.51–11.49) vs. 8.52 (5.14–12.75) ng/g, *p* = 0.029]. No significant differences were observed for other POPs between the two groups (Table 1). Spearman correlation coefficients among the 15 POPs are presented in Table 3.

**Table 3:** Isotope-labeled internal standards used for quantification.

Compound	p,p’-DDE	p,p’-DDT	o,p’-DDT	HCB	β-HCH	Heptachlor	PFOS	PFOA	PFHxS	PFNA	PFDA	PFUnDA	PFDoDA	PFBS	PFHpA
p,p’-DDE	1.000	—	—	—	—	—	—	—	—	—	—	—	—	—	—
p,p’-DDT	−0.081	1.000	—	—	—	—	—	—	—	—	—	—	—	—	—
o,p’-DDT	0.046	−0.054	1.000	—	—	—	—	—	—	—	—	—	—	—	—
HCB	−0.040	0.042	−0.067	1.000	—	—	—	—	—	—	—	—	—	—	—
β-HCH	−0.024	0.045	0.036	−0.024	1.000	—	—	—	—	—	—	—	—	—	—
Heptachlor	0.056	0.055	−0.021	−0.008	0.037	1.000	—	—	—	—	—	—	—	—	—
PFOS	0.108*	−0.050	−0.018	0.004	0.039	0.028	1.000	—	—	—	—	—	—	—	—
PFOA	−0.026	0.001	0.012	0.000	0.077	0.044	0.056	1.000	—	—	—	—	—	—	—
PFHxS	0.079	−0.065	0.027	0.050	0.012	−0.041	0.044	0.100*	1.000	—	—	—	—	—	—
PFNA	−0.074	0.019	0.005	0.043	0.031	0.150**	−0.029	−0.026	0.042	1.000	—	—	—	—	—
PFDA	0.017	0.094	0.051	0.009	−0.037	−0.020	0.071	−0.035	0.042	0.071	1.000	—	—	—	—
PFUnDA	0.030	0.022	−0.033	−0.040	−0.060	0.029	0.000	−0.062	−0.089	0.026	0.063	1.000	—	—	—
PFDoDA	−0.087	0.078	0.051	0.004	0.052	−0.020	0.006	0.068	−0.003	0.067	−0.002	0.086	1.000	—	—
PFBS	0.036	−0.074	−0.051	−0.033	−0.075	0.077	0.021	−0.065	−0.000	−0.058	0.054	0.002	−0.079	1.000	—
PFHpA	0.082	0.009	−0.070	−0.003	−0.021	0.049	−0.078	0.052	0.019	−0.053	−0.000	−0.003	−0.045	0.021	1.000

Pairwise correlations among organochlorine pesticides (OCPs) and per- and polyfluoroalkyl substances (PFAS) were assessed using Spearman’s rank correlation analysis. Values indicate correlation coefficients (r). Statistical significance is denoted as *p < 0.05 and **p < 0.01.

### 3.3 Associations between individual POPs and PCOS risk

**Figure 1:**
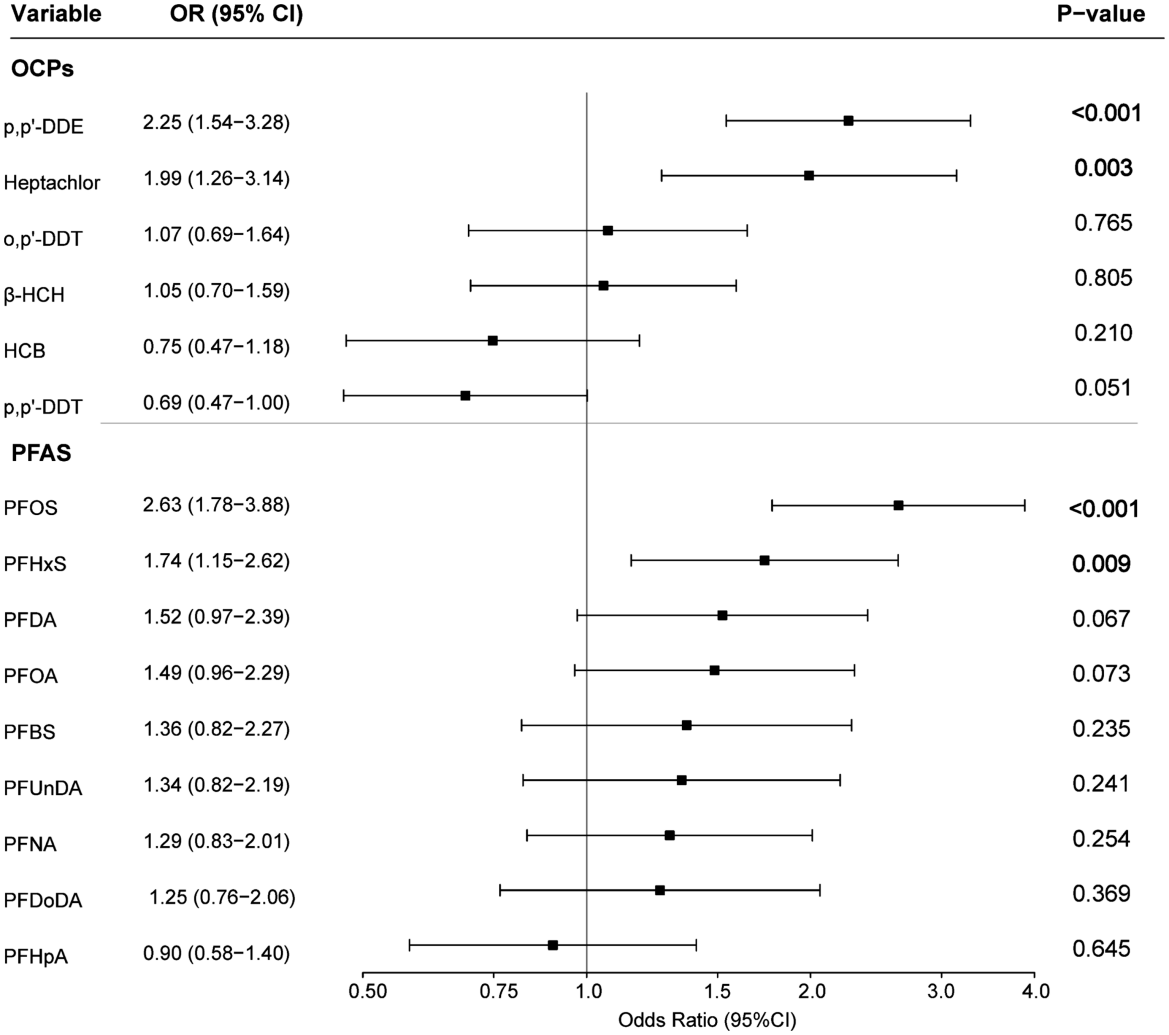
Forest plot of associations between individual POPs and PCOS risk. Adjusted odds ratios (ORs) and 95% confidence intervals (CIs) for PCOS per one-unit increase in natural log-transformed POPs concentrations. Models were adjusted for age, BMI, education, marital status, occupation, smoking, alcohol consumption, income, menarche age, gravidity, and parity. The vertical dashed line indicates OR = 1 (null effect). OCP concentrations are expressed as ng/g lipid; PFAS concentrations are expressed as ng/mL. OR, odds ratio; CI, confidence interval; OCPs, organochlorine pesticides; PFAS, per- and polyfluoroalkyl substances.

The associations between individual POPs and PCOS risk from multivariable logistic regression analyses are shown in Figure 1. After adjusting for age, BMI, education, marital status, occupation, smoking, alcohol consumption, income, menarche age, gravidity, and parity, four POPs were significantly associated with increased PCOS risk. PFOS showed the strongest association with PCOS (OR = 2.63, 95% CI: 1.78–3.88, *p* < 0.001), followed by p,p’-DDE (OR = 2.25, 95% CI: 1.54–3.28, *p* < 0.001), heptachlor (OR = 1.99, 95% CI: 1.26–3.14, *p* = 0.003), and PFHxS (OR = 1.74, 95% CI: 1.15–2.62, *p* = 0.009). No significant associations were observed for the remaining 11 POPs (all *p* > 0.05, Figure 1). Detailed crude and adjusted ORs are provided in Supplementary Table S4. Mixture model weights and BKMR posterior inclusion probabilities are presented in Tables 4, 5.

**Table 4:** Mixture analysis results: WQS weights and overall mixture effects.

A. Weighted quantile sum (WQS) regression weights				
Compound	Class	Weight	Rank	Important contributor^a^
PFOS	PFAS	0.176	1	Yes
p,p’-DDE	OCP	0.155	2	Yes
PFDA	PFAS	0.141	3	Yes
Heptachlor	OCP	0.09	4	Yes
PFNA	PFAS	0.081	5	Yes
PFHxS	PFAS	0.078	6	Yes
β-HCH	OCP	0.065	7	No
PFUnDA	PFAS	0.058	8	No
PFOA	PFAS	0.042	9	No
HCB	OCP	0.038	10	No
PFDoDA	PFAS	0.028	11	No
o,p’-DDT	OCP	0.021	12	No
p,p’-DDT	OCP	0.015	13	No
PFBS	PFAS	0.008	14	No
PFHpA	PFAS	0.004	15	No

B. Overall mixture effects on PCOS risk		
Method	OR (95% CI)	*p* value
WQS regression	9.29 (3.38–25.50)	<0.001
Quantile g-computation	7.04 (2.79–17.80)	<0.001

a Chemicals with weight > 1/15 (0.067) were considered important contributors.

All models adjusted for age, BMI, education, marital status, occupation, smoking, drinking, income, menarche age, gravidity, and parity.

**Table 5:** Bayesian kernel machine regression (BKMR) posterior inclusion probabilities.

Compound	Class	PIP	Importance^a^
PFOS	PFAS	1	High
p,p’-DDE	OCP	0.998	High
Heptachlor	OCP	0.868	High
PFNA	PFAS	0.773	High
PFHxS	PFAS	0.771	High
PFDA	PFAS	0.654	High
β-HCH	OCP	0.423	Low
PFUnDA	PFAS	0.387	Low
PFOA	PFAS	0.312	Low
HCB	OCP	0.278	Low
PFDoDA	PFAS	0.198	Low
o,p’-DDT	OCP	0.156	Low
p,p’-DDT	OCP	0.134	Low
PFBS	PFAS	0.089	Low
PFHpA	PFAS	0.067	Low

a High: PIP > 0.5; Low: PIP ≤ 0.5.

Model fitted with 10,000 MCMC iterations using Gaussian kernel, adjusted for all covariates.

### 3.4 Mixture effects of POPs on PCOS risk

**Figure 2:**
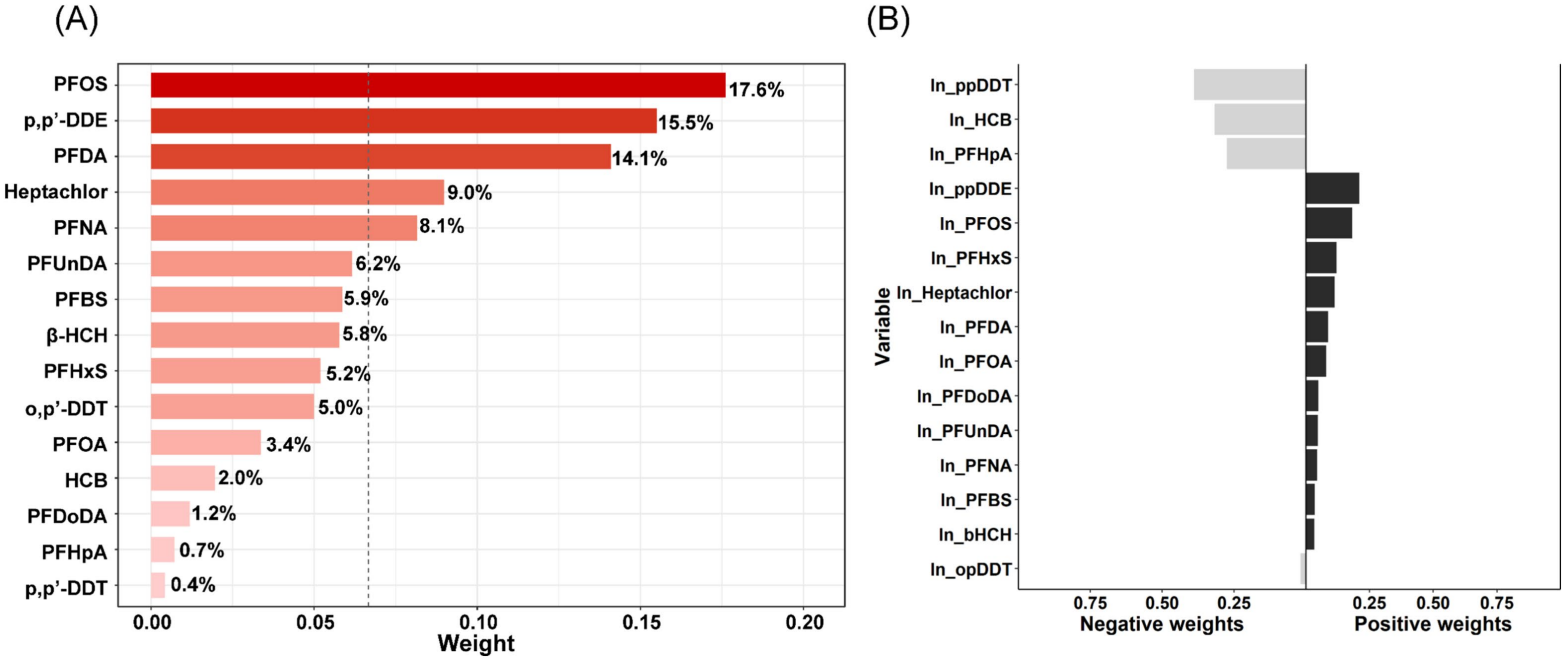
Mixture effects of POPs on PCOS risk using WQS and qgcomp models. **(A)** Weighted Quantile Sum (WQS) regression model showing the overall mixture effect and individual chemical weights. **(B)** Quantile g-computation (qgcomp) model showing the overall mixture effect and individual chemical contributions. Both models were adjusted for age, BMI, education, marital status, occupation, smoking, alcohol consumption, income, menarche age, gravidity, and parity. Positive weights/contributions indicate chemicals associated with increased PCOS risk. Error bars represent 95% confidence intervals. WQS, weighted quantile sum; qgcomp, quantile g-computation; OR, odds ratio; CI, confidence interval.

To evaluate the joint effects of POPs mixtures on PCOS risk, we employed three complementary approaches: WQS regression, qgcomp, and BKMR. Both WQS and qgcomp models demonstrated significant positive associations between the overall POPs mixture and PCOS risk (Figure 2). The WQS model yielded an OR of 9.29 (95% CI: 3.38–25.50, *p* < 0.001) for a one-quantile increase in the weighted mixture index. Similarly, the qgcomp model showed an OR of 7.04 (95% CI: 2.79–17.80, *p* < 0.001) for a simultaneous one-quantile increase in all POPs.

In the WQS model, PFOS contributed the largest weight to the mixture effect (weight = 0.176), followed by p,p’-DDE (0.155), PFDA (0.141), heptachlor (0.090), and PFNA (0.081). The qgcomp analysis yielded consistent results, with PFOS and p,p’-DDE identified as the primary contributors to the positive mixture effect (Figure 2).

**Figure 3:**
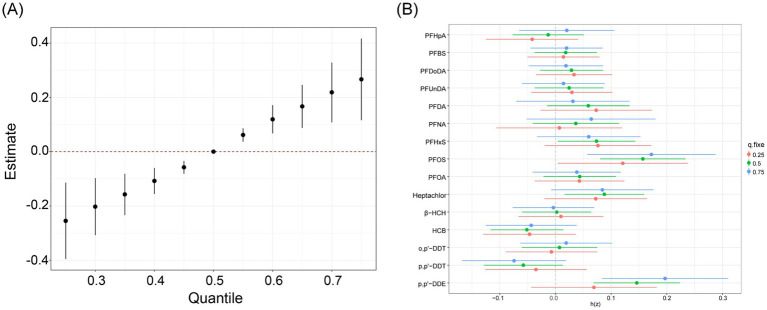
Bayesian kernel machine regression (BKMR) analysis of associations between persistent organic pollutant mixtures and PCOS risk. **(A)** Overall effect of the POPs mixture on PCOS risk. The plot shows the estimated change in the probit of PCOS probability and 95% credible intervals when all pollutants are at a particular quantile compared to when all are at the 50th percentile. **(B)** Single-pollutant effects within the mixture. The plot displays the change in PCOS risk associated with a change in each pollutant from its 25th to 75th percentile, while all other pollutants are fixed at their 25th (red), 50th (green), or 75th (blue) percentiles. Models were adjusted for age, BMI, education, marital status, occupation, smoking, alcohol consumption, income, menarche age, gravidity, and parity. BKMR, Bayesian kernel machine regression; PCOS, polycystic ovary syndrome; POPs, persistent organic pollutants.

**Figure 4:**
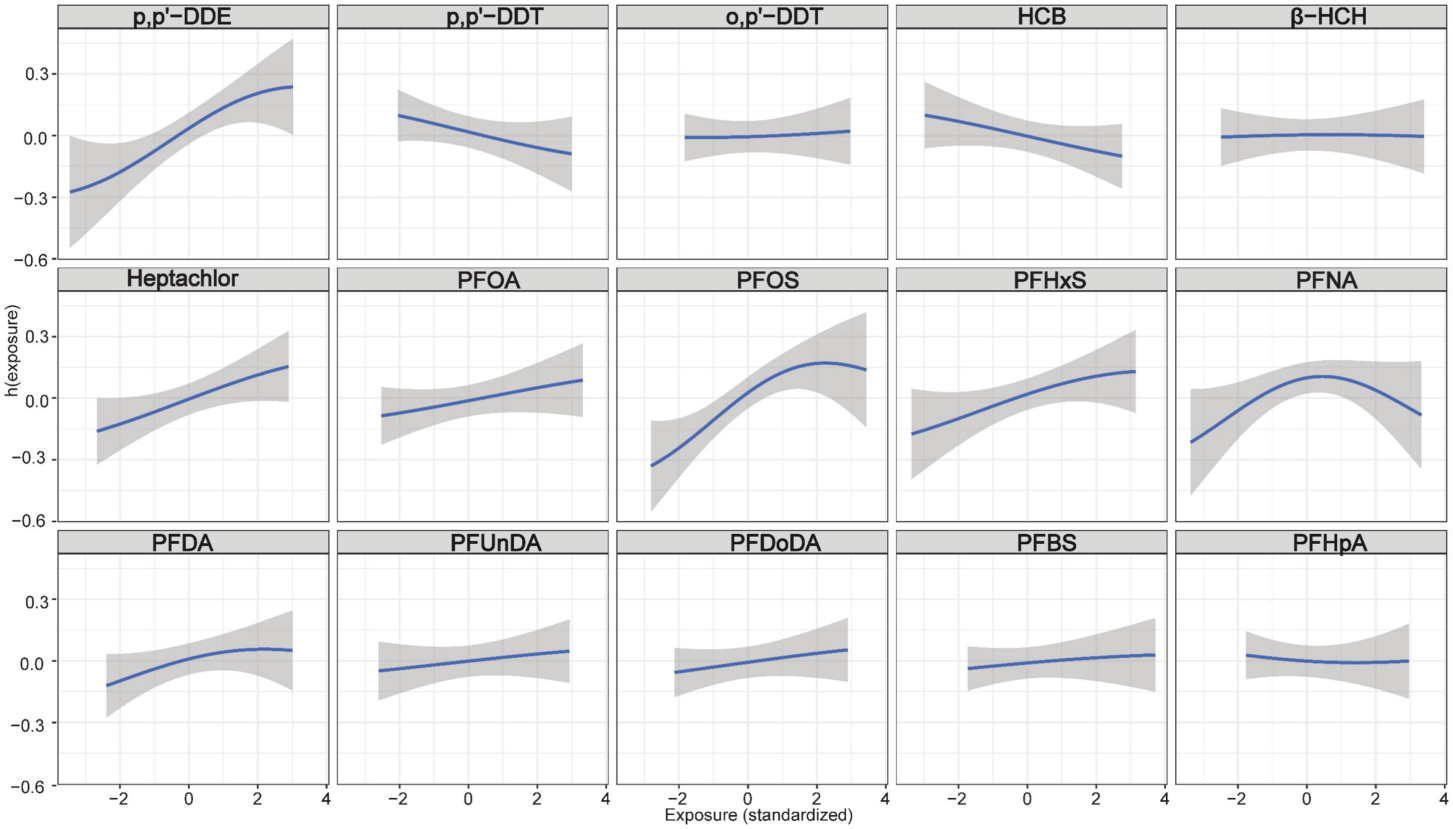
Univariate exposure-response functions for individual persistent organic pollutants and PCOS risk from BKMR analysis. The plots show the estimated exposure-response relationship between each POPs and PCOS risk, with all other pollutants fixed at their median values. The x-axis represents standardized exposure levels, and the y-axis represents the estimated change in the probit of PCOS probability. Blue lines indicate the posterior mean estimates, and grey shaded areas represent 95% credible intervals. Models were adjusted for age, BMI, education, marital status, occupation, smoking, alcohol consumption, income, menarche age, gravidity, and parity. OCP concentrations are expressed as ng/g lipid; PFAS concentrations are expressed as ng/mL. BKMR, Bayesian kernel machine regression; PCOS, polycystic ovary syndrome; POPs, persistent organic pollutants; OCPs, organochlorine pesticides; PFAS, per- and polyfluoroalkyl substances.

The BKMR analysis further confirmed these findings (Figures 3, 4). The overall mixture effect showed a dose-dependent increase in PCOS risk, with the posterior mean estimate becoming significantly positive when all pollutants exceeded the 55th percentile (Figure 3A). The posterior inclusion probabilities (PIPs) identified PFOS (PIP = 1.000) and p,p’-DDE (PIP = 0.998) as the most important contributors to the mixture effect, followed by heptachlor (PIP = 0.868), PFNA (PIP = 0.773), and PFHxS (PIP = 0.771) (Figure 3B). Notably, the single-pollutant effects remained consistent regardless of whether other pollutants were fixed at their 25th, 50th, or 75th percentiles, suggesting minimal interactions among POPs in their associations with PCOS risk (Figure 3B).

The univariate exposure-response functions revealed predominantly linear relationships between most POPs and PCOS risk (Figure 4). Positive dose–response relationships were observed for p,p’-DDE, heptachlor, PFOS, and PFHxS, while inverse or null associations were observed for p,p’-DDT, HCB, and several short-chain PFAS (Figure 4).

### 3.5 Mediation analysis of sex hormones

To explore whether sex hormones mediate the associations between POPs and PCOS, we conducted mediation analyses for the four POPs that showed significant associations with PCOS in single-pollutant models (Table 6). Using stringent criteria requiring all three components (ACME, ADE, and total effect) to be statistically significant, we identified three significant mediation effects.

**Table 6:** Mediation analysis of the associations between POPs, sex hormones, and PCOS.

Exposure	Mediator	ACME (95% CI)	*p*	ADE (95% CI)	*p*	Total effect (95% CI)	*p*	Proportion
p,p’-DDE	FSH	0.0002 (−0.0001, 0.0015)	0.194	0.0108 (0.0018, 0.0269)	**<0.001**	0.0112 (0.0018, 0.0290)	**<0.001**	1.80%
p,p’-DDE	LH	0.0071 (0.0004, 0.0347)	**<0.001**	0.0040 (−0.0006, 0.0108)	0.066	0.0134 (0.0015, 0.0348)	**<0.001**	53.00%
p,p’-DDE	E2	0.0001 (−0.0004, 0.0012)	0.47	0.0104 (0.0016, 0.0282)	**<0.001**	0.0106 (0.0016, 0.0284)	**<0.001**	0.90%
p,p’-DDE	Testosterone	0.0125 (0.0009, 0.0535)	**<0.001**	0.0008 (−0.0107, 0.0030)	0.618	0.0141 (0.0016, 0.0353)	**<0.001**	88.50%
p,p’-DDE	Prolactin	0.0005 (−0.0001, 0.0040)	0.132	0.0087 (0.0014, 0.0266)	**<0.001**	0.0099 (0.0016, 0.0292)	**<0.001**	5.30%
p,p’-DDE	Progesterone	0.0029 (0.0002, 0.0135)	**<0.001**	0.0065 (0.0007, 0.0160)	**0.002**	0.0119 (0.0013, 0.0295)	**<0.001**	24.80%
PFOS	FSH	0.0003 (−0.0036, 0.0034)	0.816	0.0851 (0.0674, 0.0980)	**<0.001**	0.0857 (0.0687, 0.0973)	**<0.001**	0.40%
PFOS	LH	0.0516 (0.0310, 0.0899)	**<0.001**	0.0240 (−0.0041, 0.0447)	0.088	0.0872 (0.0705, 0.1081)	**<0.001**	59.20%
PFOS	E2	0.0013 (−0.0006, 0.0059)	0.258	0.0827 (0.0645, 0.0958)	**<0.001**	0.0850 (0.0679, 0.0972)	**<0.001**	1.60%
PFOS	Testosterone	0.0450 (0.0273, 0.0755)	**<0.001**	0.0329 (0.0118, 0.0555)	**0.006**	0.0912 (0.0748, 0.1138)	**<0.001**	49.30%
PFOS	Prolactin	0.0042 (−0.0035, 0.0138)	0.24	0.0761 (0.0582, 0.0965)	**<0.001**	0.0834 (0.0687, 0.0991)	**<0.001**	5.10%
PFOS	Progesterone	0.0189 (0.0075, 0.0343)	**<0.001**	0.0624 (0.0405, 0.0785)	**<0.001**	0.0905 (0.0701, 0.1015)	**<0.001**	20.90%
Heptachlor	FSH	0.0010 (−0.0049, 0.0062)	0.712	0.0757 (0.0219, 0.0939)	**0.026**	0.0771 (0.0204, 0.0940)	**0.022**	1.30%
Heptachlor	LH	0.0335 (0.0009, 0.0648)	**0.05**	0.0353 (−0.0232, 0.0683)	0.196	0.0727 (0.0051, 0.1008)	**0.044**	46.10%
Heptachlor	E2	−0.0017 (−0.0107, 0.0051)	0.612	0.0786 (0.0258, 0.0952)	**0.02**	0.0763 (0.0223, 0.0934)	**0.02**	−2.20%
Heptachlor	Testosterone	0.0510 (0.0086, 0.0842)	**0.028**	0.0180 (−0.0378, 0.0511)	0.472	0.0714 (−0.0014, 0.1038)	**0.058**	71.40%
Heptachlor	Prolactin	0.0041 (−0.0109, 0.0189)	0.584	0.0694 (0.0230, 0.0924)	**0.018**	0.0748 (0.0281, 0.0945)	**0.016**	5.50%
Heptachlor	Progesterone	0.0258 (0.0045, 0.0489)	**0.016**	0.0500 (−0.0159, 0.0749)	0.112	0.0810 (0.0185, 0.0984)	**0.024**	31.90%
PFHxS	FSH	0.0004 (−0.0090, 0.0087)	0.902	0.0912 (0.0151, 0.1662)	**0.014**	0.0917 (0.0149, 0.1675)	**0.018**	0.40%
PFHxS	LH	0.0365 (−0.0078, 0.0856)	0.102	0.0615 (−0.0096, 0.1232)	0.11	0.0999 (0.0196, 0.1718)	**0.012**	36.60%
PFHxS	E2	0.0010 (−0.0085, 0.0121)	0.842	0.0929 (0.0165, 0.1720)	**0.01**	0.0940 (0.0177, 0.1723)	**0.012**	1.10%
PFHxS	Testosterone	0.0734 (0.0290, 0.1185)	**0.002**	0.0153 (−0.0471, 0.0749)	0.614	0.0895 (0.0126, 0.1633)	**0.022**	82.00%
PFHxS	Prolactin	0.0082 (−0.0106, 0.0294)	0.374	0.0762 (0.0042, 0.1531)	**0.038**	0.0854 (0.0104, 0.1642)	**0.028**	9.60%
PFHxS	Progesterone	0.0341 (0.0036, 0.0671)	**0.024**	0.0564 (−0.0115, 0.1286)	0.124	0.0925 (0.0186, 0.1696)	**0.006**	36.80%

All models were adjusted for age, BMI, education, marital status, occupation, smoking, alcohol consumption, income, menarche age, gravidity, and parity. POPs concentrations and sex hormone levels were natural log-transformed. Bootstrap method with 500 iterations was used to estimate 95% confidence intervals. ACME, average causal mediation effect (indirect effect); ADE, average direct effect; CI, confidence interval; LH, luteinizing hormone; PCOS, polycystic ovary syndrome; POPs, persistent organic pollutants. Bold values indicate statistical significance (*p* < 0.05).

For PFOS, testosterone significantly mediated 49.3% of its association with PCOS (ACME = 0.0450, 95% CI: 0.0273–0.0755, *p* < 0.001; ADE = 0.0329, 95% CI: 0.0118–0.0555, *p* = 0.006), while progesterone mediated an additional 20.9% (ACME = 0.0189, 95% CI: 0.0075–0.0343, *p* < 0.001; ADE = 0.0624, 95% CI: 0.0405–0.0785, *p* < 0.001). For p,p’-DDE, progesterone mediated 24.8% of its association with PCOS (ACME = 0.0029, 95% CI: 0.0002–0.0135, *p* < 0.001; ADE = 0.0065, 95% CI: 0.0007–0.0160, *p* = 0.002).

Although several other mediation effects showed significant indirect effects (ACME), they did not meet our strict criteria as their direct effects (ADE) were not statistically significant (Table 6). For instance, while testosterone showed significant indirect effects for p,p’-DDE (proportion mediated = 88.5%), heptachlor (71.4%), and PFHxS (82.0%), the corresponding direct effects were not significant, suggesting that the associations between these POPs and PCOS may be largely explained through their effects on testosterone levels.

## 4 Discussion

In this case–control study of Chinese women, we comprehensively investigated the associations between serum concentrations of 15 POPs and PCOS risk using both single-pollutant and mixture analysis approaches. Our findings revealed that exposure to POPs, both individually and as mixtures, was significantly associated with increased PCOS risk. In single-pollutant analyses, PFOS exhibited the strongest association with PCOS, followed by p,p’-DDE, heptachlor, and PFHxS. Mixture analyses using WQS, qgcomp, and BKMR consistently identified PFOS and p,p’-DDE as the predominant contributors to the overall mixture effect. Furthermore, mediation analyses suggested that testosterone and progesterone may partially mediate the associations between specific POPs and PCOS. To our knowledge, this is among the first studies to simultaneously evaluate multiple classes of POPs in relation to PCOS risk and to explore the potential mediating role of sex hormones in these associations.

Our finding that PFOS was the strongest contributor to PCOS risk is consistent with several previous epidemiological studies. Vagi et al. (31) first reported that women with PFOS concentrations in the highest tertile were 5.8 times more likely to have PCOS compared to those in the lowest tertile in a case–control study from Los Angeles. A recent study of women attending a U.S. fertility clinic found that per doubling of serum PFOS concentration was associated with higher odds of PCOS (OR = 1.70, 95% CI: 1.06–2.81) (16). However, the magnitude of association observed in our study (OR = 2.63) was notably higher than these previous reports, which may be attributable to differences in study populations, exposure levels, or covariate adjustment strategies. The PFOS concentrations in our study population (median: 10.24 ng/mL) were comparable to those reported in Chinese populations (median: 8.5–15.2 ng/mL) (32) but higher than those in Western populations (median: 4.0–6.0 ng/mL) (33).

We also observed a significant positive association between PFHxS and PCOS risk. A Swedish cohort study found that women with the highest estimated PFAS exposure (dominated by PFOS and PFHxS from contaminated drinking water) had a twofold increased risk for PCOS (HR = 2.18, 95% CI: 1.43–3.34) (34). Similarly, Zhang et al. (16) reported that PFHxS was associated with PCOS risk (OR = 1.45, 95% CI: 1.02–2.08) in a U.S. fertility clinic population. In contrast, we did not find significant associations for PFOA or other PFAS compounds, which is inconsistent with some previous studies (35). These discrepancies may reflect differences in exposure sources, as PFAS contamination patterns vary considerably across geographic regions and time periods.

Our study found that p,p’-DDE, the primary metabolite of DDT, was significantly associated with increased PCOS risk. Although DDT has been banned in most countries since the 1970s, its metabolite p,p’-DDE persists in the environment and human tissues due to its long half-life (approximately 7–10 years) (36). Previous studies on the relationship between OCPs and PCOS have yielded mixed results. Yang et al. (19) found that several organic pollutants, including OCPs, were significantly higher in women with PCOS compared to controls in a Chinese population. Interestingly, we observed that heptachlor, a cyclodiene insecticide historically used for termite control, was positively associated with PCOS risk. To our knowledge, this is the first study to report this association. Heptachlor has been shown to possess estrogenic and anti-androgenic properties in experimental studies (37), which may provide biological plausibility for its potential role in PCOS development.

A notable strength of our study is the application of multiple mixture analysis methods to evaluate the joint effects of POPs on PCOS risk. Traditional single-pollutant approaches may underestimate the health effects of environmental exposures because humans are simultaneously exposed to complex chemical mixtures in real-world settings (25, 26). Our WQS and qgcomp analyses demonstrated that the overall POPs mixture was strongly associated with PCOS risk, with ORs of 9.29 and 7.04, respectively. These effect estimates were substantially larger than those observed for individual pollutants, suggesting potential additive effects among co-occurring POPs. The WQS regression method was originally developed to characterize the effects of correlated environmental mixtures in risk analysis settings (27), while qgcomp provides an alternative approach that does not require constraints on the direction of individual exposure effects (29).

The BKMR analysis provided additional insights by allowing flexible, non-linear exposure-response relationships and potential interactions among pollutants (28, 38). BKMR uses a kernel machine approach to estimate the multivariable exposure-response function in a flexible and parsimonious way, while conducting variable selection on the vector of exposures (38). The overall mixture effect showed a clear dose-dependent pattern, with PCOS risk increasing monotonically as pollutant levels increased from low to high quantiles. Importantly, all three mixture methods consistently identified PFOS and p,p’-DDE as the primary drivers of the mixture effect, enhancing confidence in these findings. The parallel curves observed in the single-pollutant effects analysis (Figure 3B) suggested minimal interactions among POPs, indicating that their effects on PCOS risk are largely additive rather than synergistic. In addition, it is important to consider the potential exposure pathways through which these persistent pollutants enter the human body. Organochlorine pesticides (OCPs) are lipophilic compounds that tend to bioaccumulate in the food chain, and dietary intake—particularly through contaminated fish, meat, and dairy products—is considered the primary route of human exposure. Although many OCPs have been banned for decades, their persistence in soil and sediments allows them to remain detectable in food and human tissues. In contrast, per- and polyfluoroalkyl substances (PFAS) are widely used in industrial processes and consumer products such as non-stick cookware, food packaging materials, and water-repellent textiles. Human exposure to PFAS occurs mainly through contaminated drinking water, dietary intake, household dust, and contact with consumer products. Because both OCPs and PFAS are highly persistent and bioaccumulative, serum concentrations are widely regarded as reliable biomarkers reflecting long-term environmental exposure.

The biological mechanisms through which POPs may influence PCOS development likely involve disruption of the hypothalamic–pituitary-gonadal (HPG) axis and direct effects on ovarian steroidogenesis (23). Both PFAS and OCPs are recognized endocrine-disrupting chemicals (EDCs) that can interfere with hormone synthesis, metabolism, and receptor signaling (7). Our mediation analyses provided empirical support for the role of sex hormones in the POPs-PCOS relationship.

We found that testosterone significantly mediated 49.3% of the PFOS-PCOS association. This finding is biologically plausible, as PFOS has been shown to enhance testosterone synthesis in ovarian theca cells by upregulating steroidogenic enzymes such as CYP17A1 (22). Hyperandrogenism is a cardinal feature of PCOS, and elevated testosterone levels contribute to follicular arrest, anovulation, and the characteristic polycystic ovarian morphology (21). Additionally, progesterone mediated 20.9% of the PFOS-PCOS association and 24.8% of the p,p’-DDE-PCOS association. Progesterone deficiency, resulting from anovulation and luteal phase insufficiency, is commonly observed in women with PCOS (20).

Interestingly, although testosterone showed large mediation proportions for p,p’-DDE (88.5%), heptachlor (71.4%), and PFHxS (82.0%), the direct effects were not statistically significant. This pattern suggests that the associations between these POPs and PCOS may be predominantly mediated through their effects on testosterone, with minimal direct effects independent of this hormone. These findings underscore the central role of androgen dysregulation in mediating the reproductive toxicity of POPs. The mediation analysis approach we employed follows the counterfactual framework for causal mediation, which provides valid estimates of natural direct and indirect effects under explicitly stated assumptions (39, 40).

Our study has several notable strengths. First, we simultaneously measured 15 POPs encompassing both OCPs and PFAS, enabling comprehensive evaluation of multiple pollutant classes within a single study population. Second, we employed multiple complementary statistical approaches—single-pollutant regression, WQS, qgcomp, and BKMR—to evaluate both individual and joint effects of POPs, which enhances the robustness and credibility of our findings (25). Third, we performed mediation analyses to explore potential biological mechanisms, providing insights into how POPs may contribute to PCOS development through effects on sex hormones. Fourth, PCOS cases were diagnosed according to the Rotterdam criteria by experienced gynecologists, ensuring diagnostic accuracy. Fifth, all POPs were measured using validated GC–MS/MS and UPLC-MS/MS methods with rigorous quality control procedures.

Several limitations should be acknowledged. First, the case–control design precludes establishing causal relationships between POPs exposure and PCOS. Serum POPs concentrations were measured at a single time point after PCOS diagnosis, and we cannot rule out the possibility that PCOS-related metabolic alterations may have influenced pollutant accumulation or elimination, leading to reverse causation (41, 42). Prospective cohort studies with repeated exposure measurements are needed to establish temporality. Second, although we adjusted for multiple potential confounders, residual confounding from unmeasured factors such as dietary habits, physical activity, and genetic susceptibility cannot be excluded. In particular, dietary intake represents an important exposure pathway for persistent organic pollutants, especially organochlorine pesticides that bioaccumulate in the food chain. The lack of detailed dietary information in our dataset prevented adjustment for specific dietary patterns, which may have influenced individual exposure levels (43). Third, our study population was recruited from a single medical center, which may limit the generalizability of our findings to other populations with different exposure profiles or genetic backgrounds. Fourth, OCP concentrations were lipid-adjusted using the Phillips formula based on enzymatically measured total cholesterol and triglycerides, which may introduce estimation error compared to gravimetric lipid determination. Fifth, the mediation analysis assumes no unmeasured confounding of the mediator-outcome relationship, which may not hold in observational studies (39). In addition, the timing of blood sampling relative to the menstrual cycle was not standardized between participants. Because sex hormone concentrations may vary across the menstrual cycle, this could introduce measurement variability. However, this influence may be limited because many women with PCOS experience irregular cycles and progesterone levels are often consistently low due to chronic anovulation. POP concentrations were measured at a single time point. However, many POPs have long biological half-lives and strong bioaccumulation properties, meaning that serum concentrations are generally considered reliable indicators of long-term exposure. Nevertheless, some degree of exposure misclassification cannot be ruled out.

In conclusion, our study provides evidence that exposure to POPs, particularly PFOS and p,p’-DDE, is associated with increased PCOS risk among Chinese women. The mixture analyses revealed substantial joint effects of POPs that exceeded the sum of individual effects, highlighting the importance of considering cumulative exposures in risk assessment. The mediation analyses suggest that POPs may contribute to PCOS development partly through disruption of sex hormone homeostasis, particularly by elevating testosterone and reducing progesterone levels. These findings add to the growing body of evidence linking environmental EDC exposure to reproductive disorders and underscore the need for continued efforts to reduce human exposure to persistent organic pollutants. Future prospective studies with repeated exposure measurements and larger sample sizes are warranted to confirm these associations and further elucidate the underlying mechanisms.

## Data Availability

The original contributions presented in the study are included in the article/Supplementary material, further inquiries can be directed to the corresponding author.

## References

[1] Rotterdam ESHRE/ASRM-Sponsored PCOS Consensus Workshop Group. Revised 2003 consensus on diagnostic criteria and long-term health risks related to polycystic ovary syndrome. Fertil Steril. (2004) 81:19–25. doi: 10.1016/j.fertnstert.2003.10.004, 14711538

[2] World Health Organization. Polycystic Ovary Syndrome. World Health Organization. (2023). Available online at: https://www.who.int/news-room/fact-sheets/detail/polycystic-ovary-syndrome (Accessed October 1, 2025).

[3] Teede HJ Tay CT Laven JJE Dokras A Moran LJ Piltonen TT . Recommendations from the 2023 international evidence-based guideline for the assessment and management of polycystic ovary syndrome. Eur J Endocrinol. (2023) 189:G43–64. doi: 10.1093/ejendo/lvad096, 37580861

[4] Ferrari AJ Santomauro DF Aali A Abate YH Abbafati C Abbastabar H . Global incidence, prevalence, years lived with disability (YLDs), disability-adjusted life-years (DALYs), and healthy life expectancy (HALE) for 371 diseases and injuries in 204 countries and territories and 811 subnational locations, 1990–2021: a systematic analysis for the global burden of disease study 2021. Lancet. (2024) 403:2133–61. doi: 10.1016/s0140-6736(24)00757-8, 38642570 PMC11122111

[5] Joham AE Norman RJ Stener-Victorin E Legro RS Franks S Moran LJ . Polycystic ovary syndrome. Lancet Diabetes Endocrinol. (2022) 10:668–80. doi: 10.1016/s2213-8587(22)00163-2, 35934017

[6] Dapas M Lin FTJ Nadkarni GN Sisk R Legro RS Urbanek M . Distinct subtypes of polycystic ovary syndrome with novel genetic associations: an unsupervised, phenotypic clustering analysis. PLoS Med. (2020) 17:e1003132. doi: 10.1371/journal.pmed.1003132, 32574161 PMC7310679

[7] Gore AC Chappell VA Fenton SE Flaws JA Nadal A Prins GS . EDC-2: the Endocrine Society’s second scientific statement on endocrine-disrupting chemicals. Endocr Rev. (2015) 36:E1–E150. doi: 10.1210/er.2015-1010, 26544531 PMC4702494

[8] Diamanti-Kandarakis E Bourguignon JP Giudice LC Hauser R Prins GS Soto AM . Endocrine-disrupting chemicals: an Endocrine Society scientific statement. Endocr Rev. (2009) 30:293–342. doi: 10.1210/er.2009-0002, 19502515 PMC2726844

[9] Bonefeld-Jørgensen EC Ghisari M Wielsøe M Bjerregaard-Olesen C Kjeldsen LS Long M. Biomonitoring and hormone-disrupting effect biomarkers of persistent organic pollutants in vitro and ex vivo. Basic Clin Pharmacol Toxicol. (2014) 115:118–28. doi: 10.1111/bcpt.12263, 24797035 PMC4270256

[10] Kahn LG Harley KG Siegel EL Zhu Y Factor-Litvak P Porucznik CA . Persistent organic pollutants and couple fecundability: a systematic review. Hum Reprod Update. (2021) 27:339–66. doi: 10.1093/humupd/dmaa037 33147335 PMC7903116

[11] Mrema EJ Rubino FM Brambilla G Moretto A Tsatsakis AM Colosio C. Persistent organochlorinated pesticides and mechanisms of their toxicity. Toxicology. (2013) 307:74–88. doi: 10.1016/j.tox.2012.11.015, 23219589

[12] Wong MH Leung AOW Chan JKY Choi MPK. A review on the usage of POP pesticides in China, with emphasis on DDT loadings in human milk. Chemosphere. (2005) 60:740–52. doi: 10.1016/j.chemosphere.2005.04.028, 15949838

[13] Fenton SE Ducatman A Boobis A DeWitt JC Lau C Ng C . Per- and polyfluoroalkyl substance toxicity and human health review: current state of knowledge and strategies for informing future research. Environ Toxicol Chem. (2021) 40:606–30. doi: 10.1002/etc.4890 33017053 PMC7906952

[14] Evich MG Davis MJB McCord JP Acrey B Awkerman JA Knappe DRU . Per- and polyfluoroalkyl substances in the environment. Science. (2022) 375:eabg9065. doi: 10.1126/science.abg9065, 35113710 PMC8902460

[15] Zhan W Qiu W Ao Y Zhou W Sun Y Zhao H . Environmental exposure to emerging alternatives of per- and Polyfluoroalkyl substances and polycystic ovarian syndrome in women diagnosed with infertility: A mixture analysis. Environ Health Perspect. (2023) 131:057001. doi: 10.1289/EHP11814, 37134253 PMC10156134

[16] Zhang Y Martin L Mustieles V Ghaly M Archer M Sun Y . Per- and polyfluoroalkyl substances exposure is associated with polycystic ovary syndrome risk among women attending a fertility clinic. Sci Total Environ. (2024) 950:175313. doi: 10.1016/j.scitotenv.2024.175313, 39117221 PMC11357523

[17] Li S Li G Lin Y Sun F Zheng L Yu Y Association between perfluoroalkyl substances in follicular fluid and polycystic ovary syndrome in infertile women. Toxics. (2024);12. Available online at: https://www.mdpi.com/2305-6304/12/2/104 10.3390/toxics12020104 PMC10893032 38393199

[18] Pan W Ye X Yin S Ma X Li C Zhou J . Selected persistent organic pollutants associated with the risk of primary ovarian insufficiency in women. Environ Int. (2019) 129:51–8. doi: 10.1016/j.envint.2019.05.023 31108393

[19] Yang Q Zhao Y Qiu X Zhang C Li R Qiao J. Association of serum levels of typical organic pollutants with polycystic ovary syndrome (PCOS): a case–control study. Hum Reprod. (2015) 30:1964–73. doi: 10.1093/humrep/dev123, 26040477

[20] Azziz R Carmina E Chen Z Dunaif A Laven JSE Legro RS . Polycystic ovary syndrome. Nat Rev Dis Primers. (2016) 2:16057. doi: 10.1038/nrdp.2016.57, 27510637

[21] Rosenfield RL Ehrmann DA. The pathogenesis of polycystic ovary syndrome (PCOS): the hypothesis of PCOS as functional ovarian Hyperandrogenism revisited. Endocr Rev. (2016) 37:467–520. doi: 10.1210/er.2015-1104, 27459230 PMC5045492

[22] Chaparro-Ortega A Betancourt M Rosas P Vázquez-Cuevas FG Chavira R Bonilla E . Endocrine disruptor effect of perfluorooctane sulfonic acid (PFOS) and perfluorooctanoic acid (PFOA) on porcine ovarian cell steroidogenesis. Toxicol In Vitro. (2018) 46:86–93. doi: 10.1016/j.tiv.2017.09.030, 28982594

[23] Rutkowska AZ Diamanti-Kandarakis E. Polycystic ovary syndrome and environmental toxins. Fertil Steril. (2016) 106:948–58. doi: 10.1016/j.fertnstert.2016.08.031, 27559705

[24] Xu X Zhang X Chen J Du X Sun Y Zhan L . Exploring the molecular mechanisms by which per- and polyfluoroalkyl substances induce polycystic ovary syndrome through in silico toxicogenomic data mining. Ecotoxicol Environ Saf. (2024) 275:116251. doi: 10.1016/j.ecoenv.2024.116251, 38537477

[25] Braun JM Gennings C Hauser R Webster TF. What can epidemiological studies tell us about the impact of chemical mixtures on human health? Environ Health Perspect. (2016) 124:A6–9. doi: 10.1289/ehp.1510569 26720830 PMC4710611

[26] Carlin DJ Rider CV Woychik R Birnbaum LS. Unraveling the health effects of environmental mixtures: an NIEHS priority. Environ Health Perspect. (2013) 121:a6–8. doi: 10.1289/ehp.1206182, 23409283 PMC3553446

[27] Carrico C Gennings C Wheeler DC Factor-Litvak P. Characterization of weighted quantile sum regression for highly correlated data in a risk analysis setting. J Agric Biol Environ Stat. (2015) 20:100–20. doi: 10.1007/s13253-014-0180-3, 30505142 PMC6261506

[28] Bobb JF Valeri L Claus Henn B Christiani DC Wright RO Mazumdar M . Bayesian kernel machine regression for estimating the health effects of multi-pollutant mixtures. Biostatistics. (2015) 16:493–508. doi: 10.1093/biostatistics/kxu058, 25532525 PMC5963470

[29] Keil AP Buckley JP O’Brien KM Ferguson KK Zhao S White AJ. A quantile-based g-computation approach to addressing the effects of exposure mixtures. Environ Health Perspect. (2020) 128:047004. doi: 10.1289/EHP5838 32255670 PMC7228100

[30] Wang W Zhou W Wu S Liang F Li Y Zhang J . Perfluoroalkyl substances exposure and risk of polycystic ovarian syndrome related infertility in Chinese women. Environ Pollut. (2019) 247:824–31. doi: 10.1016/j.envpol.2019.01.039, 30731307

[31] Vagi SJ Azziz-Baumgartner E Sjödin A Calafat AM Dumesic D Gonzalez L . Exploring the potential association between brominated diphenyl ethers, polychlorinated biphenyls, organochlorine pesticides, perfluorinated compounds, phthalates, and bisphenol a in polycystic ovary syndrome: a case–control study. BMC Endocr Disord. (2014) 14:86. doi: 10.1186/1472-6823-14-86, 25348326 PMC4287339

[32] Pan Y Cui Q Wang J Sheng N Jing J Yao B . Profiles of emerging and legacy per-/Polyfluoroalkyl substances in matched serum and semen samples: new implications for human semen quality. Environ Health Perspect. (2019) 127:127005. doi: 10.1289/EHP4431, 31841032 PMC6957285

[33] Calafat AM Wong LY Kuklenyik Z Reidy JA Needham LL. Polyfluoroalkyl Chemicals in the U.S. population: data from the National Health and nutrition examination survey (NHANES) 2003–2004 and comparisons with NHANES 1999–2000. Environ Health Perspect. (2007) 115:1596–602. doi: 10.1289/ehp.10598, 18007991 PMC2072821

[34] Hammarstrand S Jakobsson K Andersson E Xu Y Li Y Olovsson M . Perfluoroalkyl substances (PFAS) in drinking water and risk for polycystic ovarian syndrome, uterine leiomyoma, and endometriosis: A Swedish cohort study. Environ Int. (2021) 157:106819. doi: 10.1016/j.envint.2021.106819, 34391986

[35] Ding N Harlow SD Randolph JF Jr Loch-Caruso R Park SK. Perfluoroalkyl and polyfluoroalkyl substances (PFAS) and their effects on the ovary. Hum Reprod Update. (2020) 26:724–52. doi: 10.1093/humupd/dmaa018, 32476019 PMC7456353

[36] Toxicological Profile for DDT, DDE, and DDD. Atlanta (GA): Agency for Toxic Substances and Disease Registry (US); (2022). Agency for Toxic Substances and Disease Registry (ATSDR) Toxicological Profiles. Available online at: http://www.ncbi.nlm.nih.gov/books/NBK590084/37023235

[37] Mnif W Hassine AIH Bouaziz A Bartegi A Thomas O Roig B. Effect of endocrine disruptor pesticides: A review. Int J Environ Res Public Health. (2011) 8:2265–303. doi: 10.3390/ijerph8062265, 21776230 PMC3138025

[38] Bobb JF Claus Henn B Valeri L Coull BA. Statistical software for analyzing the health effects of multiple concurrent exposures via Bayesian kernel machine regression. Environ Health. (2018) 17:67. doi: 10.1186/s12940-018-0413-y 30126431 PMC6102907

[39] VanderWeele TJ. Mediation analysis: A practitioner’s guide. Annu Rev Public Health. (2016) 37:17–32. doi: 10.1146/annurev-publhealth-032315-021402, 26653405

[40] Richiardi L Bellocco R Zugna D. Mediation analysis in epidemiology: methods, interpretation and bias. Int J Epidemiol. (2013) 42:1511–9. doi: 10.1093/ije/dyt127, 24019424

[41] Dhingra R Winquist A Darrow LA Klein M Steenland K. A study of reverse causation: examining the associations of Perfluorooctanoic acid serum levels with two outcomes. Environ Health Perspect. (2017) 125:416–21. doi: 10.1289/EHP273, 27529882 PMC5332181

[42] Schulz KF Grimes DA. Case-control studies: research in reverse. Lancet. (2002) 359:431–4. doi: 10.1016/S0140-6736(02)07605-5, 11844534

[43] Le Marchand L. The predominance of the environment over genes in Cancer causation: implications for genetic epidemiology. Cancer Epidemiol Biomarkers Prev. (2005) 14:1037–9. doi: 10.1158/1055-9965.EPI-04-0816, 15894649

