# Supplementary material for: Associations between serum persistent organic pollutants and polycystic ovary syndrome risk: a case–control study with mediation analysis
Source: Front Public Health. 2026 Jun 8;14:1807105. doi: 10.3389/fpubh.2026.1807105 (PMC13284083; doi:10.3389/fpubh.2026.1807105)
Supplement: Supplementary file 1 [file Presentation_1.PPTX]

## Slide 1
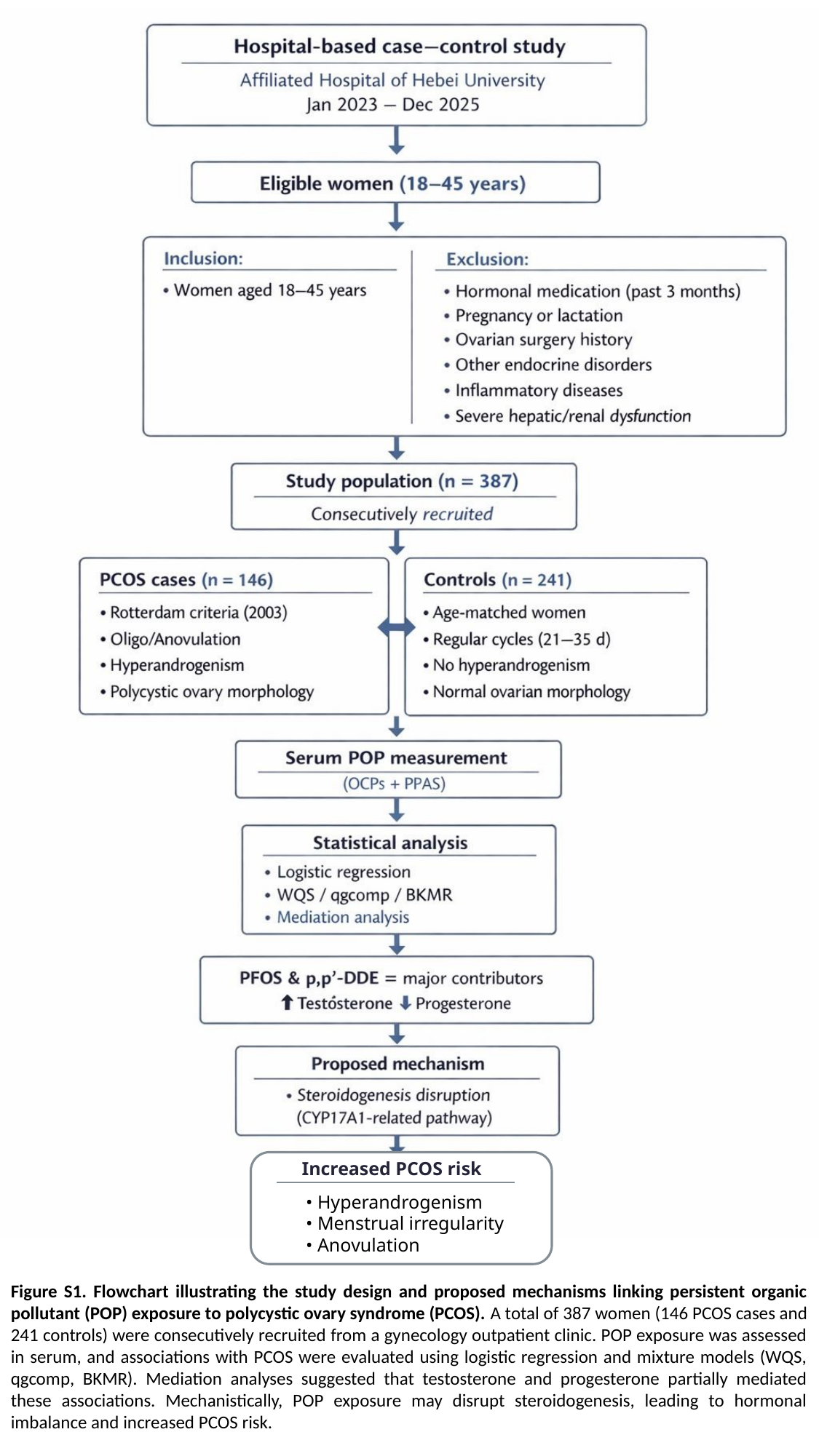

Increased PCOS risk
• Hyperandrogenism
• Menstrual irregularity
• Anovulation
Figure S1. Flowchart illustrating the study design and proposed mechanisms linking persistent organic pollutant (POP) exposure to polycystic ovary syndrome (PCOS). A total of 387 women (146 PCOS cases and 241 controls) were consecutively recruited from a gynecology outpatient clinic. POP exposure was assessed in serum, and associations with PCOS were evaluated using logistic regression and mixture models (WQS, qgcomp, BKMR). Mediation analyses suggested that testosterone and progesterone partially mediated these associations. Mechanistically, POP exposure may disrupt steroidogenesis, leading to hormonal imbalance and increased PCOS risk.
